# Deep Ensemble Learning for Multiclass Skin Lesion Classification

**DOI:** 10.3390/bioengineering12090934

**Published:** 2025-08-29

**Authors:** Tsu-Man Chiu, I-Chun Chi, Yun-Chang Li, Ming-Hseng Tseng

**Affiliations:** 1 School of Medicine, Chung Shan Medical University, Taichung 402, Taiwan; cshy2085@csh.org.tw; 2 Department of Dermatology, Chung Shan Medical University Hospital, Taichung 402, Taiwan; leeyc4372@gmail.com; 3 Department of Biomedical Sciences and Engineering, National Central University, Taoyuan 320, Taiwan; jenny901918@gmail.com; 4 Information Technology Office, Chung Shan Medical University Hospital, Taichung 402, Taiwan

**Keywords:** skin lesions, dermoscopic images, ensemble learning, CNN, ViT, Swin

## Abstract

The skin, the largest organ of the body, acts as a protective shield against external stimuli. Skin lesions, which can be the result of inflammation, infection, tumors, or autoimmune conditions, can appear as rashes, spots, lumps, or scales, or remain asymptomatic until they become severe. Conventional diagnostic approaches such as visual inspection and palpation often lack accuracy. Artificial intelligence (AI) improves diagnostic precision by analyzing large volumes of skin images to detect subtle patterns that clinicians may not recognize. This study presents a multiclass skin lesion diagnostic model developed using the CSMUH dataset, which focuses on the Eastern population. The dataset was categorized into seven disease classes for model training. A total of 25 pre-trained models, including convolutional neural networks (CNNs) and vision transformers (ViTs), were fine-tuned. The top three models were combined into an ensemble using the hard and soft voting methods. To ensure reliability, the model was tested through five randomized experiments and validated using the holdout technique. The proposed ensemble model, Swin-ViT-EfficientNetB4, achieved the highest test accuracy of 98.5%, demonstrating strong potential for accurate and early skin lesion diagnosis.

## 1. Introduction

The skin is the largest organ of the human body and plays multiple roles such as protecting, regulation of body temperature, and vitamin D production. Prolonged exposure to ultraviolet (UV) light not only causes cellular changes in the skin but is also a major cause of skin diseases. In addition to UV exposure, aging is another important factor, while other triggers include burns, radiation, genetic inheritance, chronic arsenic poisoning, and imbalanced immunity. The manifestations of skin diseases range from mild to severe and take various forms, including inflammatory, infectious, neoplastic, and autoimmune. According to the American Cancer Society’s 2024 statistics [1], skin cancer is currently the most common type of cancer, and although malignant melanoma (MM) accounts for only about 1% of all skin cancers, it is the leading cause of most skin cancer-related deaths. Moles on the skin often raise concerns due to diagnostic uncertainty. Although most moles are harmless, some lesions that appear like moles may actually be skin cancer, especially MM and Basal cell carcinoma (BCC). Examination is also necessary for other clinically suspected neoplastic lesions that may resemble MM in order to facilitate early diagnosis and treatment.

In the field of computer vision, the classification of skin lesions is a critical research topic. Due to significant variations in dermatologist clinical experience and the time-consuming nature of the diagnostic process, the development of reliable computer-aided diagnostic systems to assist in the detection and classification of skin lesions is essential to improve the quality of healthcare care. Earlier methods for skin lesion classification often involved hand-crafted feature extraction combined with machine learning algorithms [2]. Although these methods were effective in simple cases, they struggled with complex and diverse skin lesions, leading to misdiagnosis or omission. With the increase in deep learning, many studies have begun to use CNNs for the classification of skin lesions [2,3,4,5,6,7,8,9,10,11,12,13,14,15], which significantly outperforms traditional methods in terms of efficiency. Asaduzzaman et al. [15] used generative adversarial networks (GANs) to identify and remove fake images, further improving the performance of the model.

According to Table 1, which summarizes seven recent review articles on artificial intelligence in computer-aided skin lesion diagnosis, several key challenges and research directions have emerged in the field:

- Class imbalance: Many studies highlight the uneven distribution of skin lesion categories in datasets, which can lead to biased model performance.
- Lack of standardized evaluation metrics: There is a notable inconsistency in the metrics used across studies, making it difficult to compare results and assess model reliability.
- Use of ensemble techniques: Ensemble learning has gained popularity for improving classification accuracy and robustness, especially in multiclass tasks.
- Need for explainable AI: The black-box nature of deep learning models limits clinical adoption, prompting calls for interpretable and transparent systems.
- Data diversity and augmentation: To address data scarcity and improve generalization, researchers emphasize the importance of diverse datasets and augmentation strategies.
- Integration of clinical metadata: Future directions suggest combining imaging data with patient history to enhance diagnostic precision and clinical relevance.

These insights provide the foundation for our proposed ensemble framework, which aims to address these challenges through a hybrid architecture and robust evaluation strategy.

Most previous studies have focused on binary classification tasks for melanoma [16,17,18]. This study aims to develop a computer-aided diagnostic system for multiclass skin lesions using a multi-architecture ensemble framework. Ray (2018) [19] used the ResNet50 model to extract features from dermoscopic images, which are subsequently classified using the Deep Forest algorithm. The approach demonstrates improved accuracy in the classification of skin diseases, particularly in the detection of melanoma, by combining the strengths of deep learning for feature extraction and traditional ensemble methods for classification. Milton (2019) [20] presented an ensemble of deep neural networks specifically designed for the ISIC 2018 challenge, focusing on melanoma detection. The approach combines multiple deep learning models to improve robustness and accuracy, achieving competitive performance in automated skin lesion classification. Mahbod et al. (2020) [21] utilized transfer learning with a multiscale, multinetwork approach to classify skin lesions. The method demonstrates high accuracy across different datasets, showcasing the effectiveness of ensemble techniques and transfer learning in medical image analysis. Thurnhofer-Hemsi et al. (2021) [22] proposed an ensemble of deep convolutional networks combined with a regularly spaced shifting technique for the classification of skin lesions. Alshawi et al. (2023) [23] presented an ensemble learning approach that uses multiple CNNs architectures for the detection and classification of images of skin cancer. The ensemble method achieves high accuracy and robustness, making it a promising tool for automated skin cancer diagnostics.

However, the existing ensemble methods have predominantly relied on CNN-based architectures. In recent years, Vision Transformer (ViT) models have emerged as a significant advancement in computer vision tasks [18,24]. Unlike CNNs, ViTs utilize an attention mechanism that enables more effective processing of input data, particularly in large-scale image analysis. Additionally, ViTs exhibit spatial awareness, allowing them to capture spatial relationships within the data, which is an essential capability for accurate skin lesion classification.

This paper explores the use of deep ensemble learning combined with CNNs and ViTs to improve the performance of multiclass skin lesion classification. The primary objective of this study is to develop a robust and generalizable deep learning framework for the multiclass classification of skin lesions. To achieve this, the study aims to address several key challenges in dermatological image analysis:

- Examine the impact of removal of the background image on the performance of skin lesion classification.
- Explore strategies for addressing class imbalance in dermatological image datasets.
- Investigate the design of a multi-architecture ensemble model for the classification of multiple skin lesion types.

**Table 1:** Seven review articles published in the past three years.

Reference	Years	Papers	Conclusions	Future Directions
[25]	2000~2022	91	Current deep learning models are effective in dermatology but suffer from biases related to dataset limitations. Performance varies significantly depending on the different skin conditions.	Address bias in data sets by incorporating more diverse data. Integrate the clinical history with the imaging data for better diagnostics.
[26]	2015~2023	153	Machine learning algorithms have improved the diagnostic accuracy in skin diseases. There is a lack of standardized evaluation metrics in all studies.	Establish standardized benchmarks for the evaluation of machine learning models. Focus on developing interpretable models to gain the trust of the clinician.
[27]	2017~2023	89	SVMs and CNNs are the most widely used algorithms in skin lesion classification. Ensemble methods that combine different algorithms show promise.	Investigate the potential of hybrid models that combine SVMs with deep learning techniques. Improve model efficiency for real-time clinical applications.
[28]	2007~2023	37	Deep learning has advanced the automated diagnosis and monitoring of skin diseases. The challenge of ensuring the reliability and precision of these models in clinical settings remains.	Develop methods for continuous learning to adapt to new data. Focus on real-world validation of AI models in dermatology.
[29]	2001~2023	632	Progress in skin lesion segmentation and classification using machine learning. Deep learning models have shown high accuracy, but are limited by the need for large datasets.	Explore data augmentation techniques to overcome data scarcity. Develop models that can be generalized to various skin types.
[30]	1997~2024	74	Innovative machine learning approaches, such as GANs and attention mechanisms, are enhancing skin disease diagnostics. However, the clinical translation of these methods remains limited.	Explore the use of explainable AI to bridge the gap between model predictions and clinical decision making. Expand research to include underrepresented populations to improve the fairness of the model.
[31]	2017~2024	143	CNN- and ViT-based models, covering data selection, image processing, and model interpretability techniques. Despite significant progress in melanoma classification, the black-box nature of deep learning still limits clinical adoption.	Enhancing the dataset, using transfer learning, improving image quality, and integrating model interpretability methods. Large-scale clinical validation and development of explainable AI (XAI) are essential for clinical applicability.

## 2. Materials and Methods

### 2.1. CSMUH Dataset

This study used a dataset of dermoscopic image data collected by dermatologists at Chung Shan Medical University Hospital, each case confirmed by pathology. All patients in this dataset were Asian. The dataset was classified using the ISIC framework and includes melanoma (111 cases), melanocytic nevi (199 cases), basal cell carcinoma (45 cases), actinic keratosis (82 cases), benign keratosis (134 cases), dermatofibroma (42 cases) and vascular lesions (53 cases), totaling 666 cases. Information regarding the CSMUH dataset is provided in Table 2. The distribution of labels within this dataset is notably uneven, with the ratio of the maximum to the minimum number of labels reaching 4.738. This indicates a significant imbalance. To address this issue, this study will employ data augmentation techniques to achieve a more balanced distribution within the training dataset. The following is a brief description of the seven types of cutaneous neoplastic lesions of clinical interest.

Malignant Melanoma (MM): Malignant melanoma is one of the most severe and aggressive forms of skin cancer and is of great clinical importance. It tends to occur in people with high exposure to the sun, light-colored skin, and red hair, and its global incidence is increasing. According to the World Health Organization (WHO), there were 61,900 new cases of MM worldwide in 2018, resulting in 41,000 deaths.

Melanocytic Nevus (Mv): Melanocytic nevus is a common benign skin tumor formed by melanocytes. It usually develops in childhood or early childhood, and most people develop several moles throughout their lifetime. Although most moles are harmless, some may develop into MM, so it is important to monitor any new moles and seek medical evaluation.

Basal cell carcinoma (BCC): Basal cell carcinoma is the most common type of skin cancer and is the least aggressive. It usually occurs in areas with high sun exposure, such as the face, neck, and arms, and is more common in the elderly. According to the American Academy of Dermatology (AAD), there are more than 4 million new cases of basal cell carcinoma each year in the United States.

Actinic keratosis (AK): Actinic keratosis is caused by sun damage to the skin and typically appears in areas exposed to the sun such as the face and arms. Actinic keratosis can be solitary or clustered and, while most are harmless, there is a risk of progression to skin cancer, such as squamous cell carcinoma.

Benign keratosis (BK): Benign keratosis is another common benign skin tumor, usually found in older adults. It is characterized by well-demarcated, light tan to dark brown or black, oval or round, flat or elevated lesion with a rough, waxy, or “stuck-on” surface. These tumors are nonaggressive and do not turn into malignant tumors, but patients often worry that they are symptoms of skin cancer.

Dermatofibroma (DF): Dermatofibroma is a benign tumor formed by fibroblasts on the skin and usually appears in the limbs, trunk, or buttocks. These tumors are typically solitary and generally do not become malignant.

Vascular lesions (Vasc): Vascular lesions are tumors formed by blood vessels on the skin and can be congenital or acquired due to vascular abnormalities. Types of vascular lesions include keratotic angiomas, spider angiomas, and cherry angiomas. Most vascular lesions are harmless, but if changes or rapid growth occur, a doctor should be sought.

### 2.2. Research Framework

**Figure 1:**
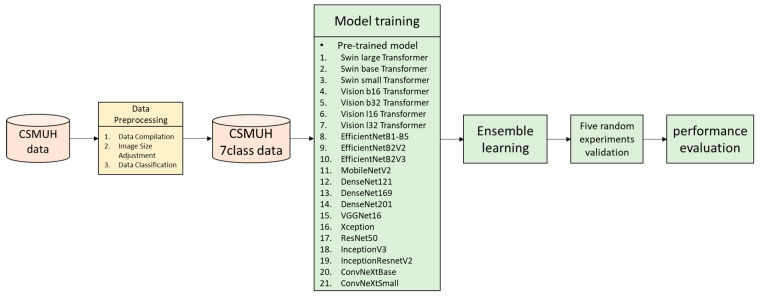
Research flowchart.

Figure 1 illustrates the step-by-step workflow of the proposed study. The model development process begins with the CSMUH dataset, which undergoes a series of preprocessing steps, including data compilation, image resizing, and classification into seven distinct skin lesion categories. This results in a structured dataset referred to as CSMUH 7-class data. Subsequently, a diverse set of pre-trained models is fine-tuned. Following individual model training, ensemble learning is employed to integrate the strengths of the top-performing models. The ensemble model is then validated through five randomized experiments to ensure robustness and consistency. Finally, model performance is evaluated using standard metrics to assess its effectiveness in skin lesion classification.

The CSMUH dataset used in this study is relatively small and exhibits significant class imbalance. Applying cross-validation under such conditions may lead to biased or unstable results due to insufficient representation of minority classes in each fold. To ensure robustness, we conducted five independent randomized experiments using a holdout validation approach. In each experiment, the dataset was randomly split into training and testing subsets with an 8:2 ratio, stratified by class distribution to maintain proportional representation. This process was repeated five times with different random seeds, and the reported accuracy and standard deviation values in all tables reflect the mean and variability across these five runs. To avoid data leakage, no images were shared between the training and testing sets, making them completely mutually exclusive. This approach allows the model to be trained on a representative portion of the data while maintaining an independent set for unbiased evaluation.

To assess the performance of the proposed model, we employed the following standard evaluation metrics:

- Accuracy: The proportion of correctly classified samples among all samples, as defined in Equation (1).
- Sensitivity (Recall): The ability of the model to correctly identify positive cases, as defined in Equation (2).
- Specificity: The ability of the model to correctly identify negative cases, as defined in Equation (3).
- Precision (PPV): The proportion of true positives among all predicted positives, as defined in Equation (4).
- Negative predictive value (NPV): The proportion of predicted negatives that are truly negative, as defined in Equation (5).
- False positive rate (FPR): The rates of incorrect predictions in positive classes, as defined in Equation (6).
- False negative rate (FNR): The rates of incorrect predictions in negative classes, as defined in Equation (7).
- F1 Score: The harmonic mean of precision and recall, providing a balanced measure of model performance, as defined in Equation (8).

where TP refers to cases where the model correctly predicts a positive outcome, TN refers to cases where the model correctly predicts a negative outcome, FP refers to cases where the model incorrectly predicts a positive outcome, and FN refers to cases where the model incorrectly predicts a negative outcome. These metrics were calculated based on the confusion matrix derived from the test set predictions.

(1) $$ACC=\frac{TP+TN}{TP+TN+FP+FN}$$

(2) $$Sensitivity=\frac{TP}{TP+FN}$$

(3) $$Specificity=\frac{TN}{TN+FP}$$

(4) $$PPV=\frac{TP}{TP+FP}$$

(5) $$NPV=\frac{TN}{TN+FN}$$

(6) $$FPR=\frac{FP}{TN+FP}$$

(7) $$FNR=\frac{FN}{TP+FN}$$

(8) $$F1\;score=\frac{2\times PPV\times Sensitivity}{PPV+Sensitivity}$$

### 2.3. Data Preprocessing

The CSMUH dataset was divided into training and testing sets, with an 8:2 split stratified by class distribution to ensure that each class was represented in both sets without any overlapping images, with all images resized to 224 × 224 pixels and normalized to facilitate computation in deep learning models. To increase the diversity of the training data, data augmentation techniques [32] such as random flipping, rotation, translation, and scaling were applied. These augmentations were applied only to the training set and not to the test set. Before model training, a static data augmentation procedure was implemented to balance the label distribution within the training dataset. For instance, the initial label distribution was (89, 159, 36, 65, 107, 34, 42). After applying static augmentation techniques, the distribution was adjusted to (178, 159, 144, 130, 107, 170, 168). In addition to static augmentation applied before training to balance class distribution, a dynamic data augmentation strategy was employed during the training phase to further diversify the input data and improve the model’s generalization capability.

### 2.4. Transfer Learning

Transfer learning involves applying models from one domain to a new domain, transferring knowledge from a source task to a target task, eliminating the need to train a model from scratch. Its advantage lies in reducing the amount of training data required. Twenty-five different pretrained models were selected, including transformer-based architectures such as Swin Transformer (small, base, large) [24] and Vision Transformer (b16, b32, l16, l32) [33], as well as CNN-based models like EfficientNet (B1–B5, V2B2, V2B3) [34,35], MobileNetV2, DenseNet (121, 169, 201), VGGNet16, Xception, ResNet50, InceptionV3, InceptionResNetV2, and ConvNeXt (Base, Small).

Based on preliminary experiments, we found that fine-tuning all layers of the pre-trained models yielded better performance than freezing the initial layers. Therefore, in our approach, all network weights were updated during training to maximize feature adaptation to the CSMUH dataset.

### 2.5. Ensemble Architecture

This study used ensemble learning techniques to improve model prediction performance by combining different models to achieve better results. The voting ensemble method was used, where the outputs of multiple independent models were combined as inputs to train a new model. In this approach, several models (sub-models) are trained independently and their predictions are combined through a voting process [36]. Each model makes predictions on each input sample, and the final output prediction is the one that receives the majority of votes among all models. This method takes advantage of the strengths of multiple learning algorithms to improve prediction performance and reduce the likelihood of errors from any single model.

This study conducted a comparative analysis of hard voting and soft voting strategies. In hard voting, each classifier outputs a discrete class label. The final prediction is determined by majority vote, where the class receiving the most votes is selected. In soft voting, each classifier provides a probability distribution over all classes. These probabilities are averaged, and the class with the highest combined probability is selected as the final prediction. In this study, all classifiers were assigned equal weights. For the final ensemble, three better classifiers were selected based on their individual performance across Swin Transformer, Vision Transformer, and CNN-based architectures.

### 2.6. Model Architecture

**Figure 2:**
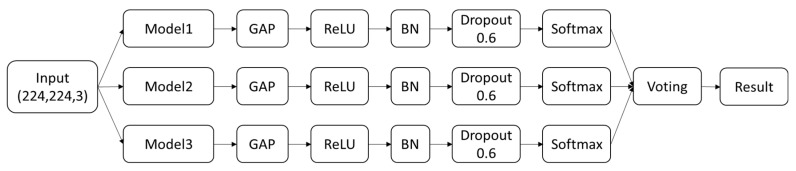
Model Architecture Diagram.

This study builds an ensemble deep learning model based on three pre-trained models, using the voting method. The top three best-performing pre-trained models were selected, followed by the addition of a fully connected layer with a ReLU activation function and a Global Average Pooling (GAP) layer. To avoid overfitting, Batch Normalization (BN) and Dropout layers were added. After obtaining the prediction results from each model for skin lesion classification, a voting process was used to determine the final prediction. The model architecture is illustrated in Figure 2.

## 3. Results

### 3.1. Effect of Removing a Dermoscopic Scale

The original images contained a dermoscopic scale, which was removed by cropping the images prior to training. Both the original and cropped images were utilized for model training. As shown in Table 3, the cropped images demonstrated superior performance in both training and testing compared to the unprocessed images. Therefore, all subsequent training and testing was conducted using the cropped images.

### 3.2. Effect of Different Pretrained Models

Table 4 presents the training and testing performance of 25 pretrained models across two architectural categories: CNN and Vision Transformer (ViT), applied to seven-class skin lesion classification. CNN models, such as EfficientNetB1 and B3, show strong training accuracy (up to 0.993), but moderate test accuracy, with EfficientNetB4 reaching 0.816. ViT models demonstrate slightly better generalization, with Vision l16 and l32 Transformers achieving a test accuracy of 0.840. Swin models, particularly Swin Large Transformer, outperform others with the highest test accuracy of 0.849, indicating superior capability in capturing both local and global features. Overall, the results suggest that Swin architectures offer a more balanced and effective approach for medical image classification, combining the strengths of CNNs and transformers to improve diagnostic reliability.

### 3.3. Effect of Hard Voting and Soft Voting Ensemble Methods

Table 5 compares the performance of different ensemble voting strategies, hard voting and soft voting, across three architectural categories: CNN, ViT, and a mixed ensemble (Swin-ViT-EfficientNetB4). Under hard voting, the best test accuracy was 0.954 from both the ViT-only ensemble and the mixed ensemble. Under soft voting, the mixed ensemble achieved the highest test accuracy of 0.961, outperforming all other configurations. CNN and ViT models performed well individually, but Swin models showed lower test accuracy, especially with hard voting (0.903).

These findings underscore the effectiveness of soft voting ensembles that integrate diverse model types. By combining CNNs (strong in local feature extraction) with transformers (effective in capturing global context), the ensemble model achieves superior generalization and robustness. This approach mitigates the limitations of individual architectures and enhances diagnostic accuracy in skin lesion classification. The results support the use of hybrid ensemble learning as a promising strategy for improving reliability in medical image analysis.

### 3.4. Effect of Different Ensemble Weights in Soft Voting Methods

Additionally, Table 6 shows the results of model training and testing with different ensemble weight combinations to evaluate their effectiveness in the classification of skin lesions. The results indicate that with a weight combination of (1, 1, 1), the model achieved the highest training and testing accuracy, reaching 0.999 ± 0.001 and 0.961 ± 0.020, respectively. This suggests that combining multiple models effectively improves prediction performance and further confirms the advantage of ensemble learning in improving model predictions.

### 3.5. Performance Indices of the Best Model

Table 7 and Table 8 present the confusion matrices of the best-performing model during the training and testing phases, respectively, while Table 9 summarizes the testing performance indices across all classes. Overall, the model demonstrated strong classification capability for most categories. Specifically, for Class 0, 3, 4, and 6, all evaluation metrics—including sensitivity, specificity, positive predictive value (PPV), negative predictive value (NPV), false positive rate (FPR), false negative rate (FNR), and F1 score—achieved optimal values, indicating exceptionally high consistency and predictive accuracy in distinguishing these classes.

For Class 1, the model also exhibited highly stable and reliable performance. The sensitivity reached 1.000, suggesting that all actual positive cases were correctly identified, while the specificity was 0.978, indicating a high level of precision in identifying negative instances. These results suggest that the model’s performance on this class remains robust and practically applicable.

In the case of Class 2 and Class 5, although the sensitivities were slightly lower (0.889 and 0.875, respectively) compared to other classes, the model still achieved excellent performance on the remaining metrics. Both classes yielded perfect specificity and PPV (1.000), and their NPV values exceeded 0.99, reflecting strong reliability in negative predictions. The corresponding F1 scores were 0.941 for Class 2 and 0.933 for Class 5, indicating that the overall predictive performance remains strong. These slightly lower sensitivities may suggest increased difficulty in distinguishing certain samples in these classes, which could be attributed to class distribution or feature similarity. This aspect warrants further investigation and potential refinement in future work.

## 4. Discussion

The results of this study demonstrate that the ensemble model significantly outperforms individual models in terms of precision and stability for the classification of skin lesions. In the CSMUH dataset, the model that combined Swin Transformer, Vision Transformer (ViT), and EfficientNetB4 showed excellent performance, successfully improving image classification accuracy. This improvement can be attributed to the complementary strengths of the integrated architectures. Convolutional Neural Networks (CNNs), such as EfficientNetB4, are highly effective at capturing local spatial features through hierarchical convolutional layers, making them well-suited for texture-rich medical images. In contrast, Vision Transformers (ViT) and Swin Transformers excel at modeling long-range dependencies and global contextual information by leveraging self-attention mechanisms. The hybrid ensemble approach benefits from both local feature extraction and global representation learning, leading to enhanced generalization and robustness. By combining CNN and transformer-based models through soft voting, the ensemble mitigates the limitations of individual architectures and achieves superior diagnostic accuracy. These findings underscore the potential of hybrid deep learning frameworks in advancing automated skin lesion classification, particularly in diverse and complex clinical datasets.

The findings of this study reveal the great potential of ensemble deep learning methods in clinical applications, particularly in improving early diagnosis and treatment outcomes for skin lesions. However, to further increase the practical value of these models, future research should focus on addressing current generalization issues and continue to explore more optimal ensemble strategies to improve the diagnostic performance of models across diverse datasets, allowing them to play a greater role in real clinical environments.

Table 10 provides a comparative result for seven-class skin lesion classification, including the authors, year, methods, validation, datasets, and overall accuracy on test sets. The proposed ensemble model, which integrates the Swin Transformer, Vision Transformer, and EfficientNetB4 with a soft voting strategy, significantly outperforms traditional CNN-based approaches. Compared to previous work [37] using the same dataset and holdout validation, the proposed ensemble model achieved the best test accuracy of 98.5%, demonstrating superior classification performance. This highlights the effectiveness of combining various architectures to enhance diagnostic accuracy in multiclass skin lesion classification tasks.

In summary, the novelty of this study is reflected in the following key aspects:

1. Multi-architecture ensemble model design: This research introduces a deep learning ensemble framework that integrates Swin Transformer, Vision Transformer (ViT), and EfficientNetB4. By employing a soft voting strategy, the model effectively combines the strengths of these three architectures, achieving a test accuracy of 98.5% in multiclass skin lesion classification—significantly outperforming individual models.
2. Optimization for multiclass classification tasks: While most prior studies have focused on binary classification of melanoma, this study addresses the classification of seven distinct skin lesion types, thereby enhancing the breadth and practical relevance of clinical applications.
3. Strong generalization capability: The proposed architecture is not tailored to a specific dataset, demonstrating robust generalizability across different datasets and clinical environments.
4. Class imbalance handling strategy: To address the uneven class distribution in the CSMUH dataset, the study employs both static and dynamic data augmentation techniques, effectively improving the model’s ability to recognize minority classes.
5. Comprehensive performance evaluation: Beyond accuracy, the study reports multiple evaluation metrics including sensitivity, specificity, positive predictive value (PPV), negative predictive value (NPV), and F1 score, underscoring the model’s stability and reliability in clinical settings.

These innovations not only enhance classification performance but also provide a practical and effective tool for early diagnosis and clinical support in dermatology.

## 5. Conclusions

This study introduces an ensemble deep learning framework that integrates Swin Transformer, Vision Transformer, and EfficientNetB4 to address the challenge of multiclass skin lesion classification. By combining the complementary strengths of convolutional neural networks and transformer-based architectures, the proposed model achieves high diagnostic accuracy, reaching 98.5% on the CSMUH dataset. This result surpasses several recent approaches. For instance, Liu et al. (2024) [38] developed SkinNet using stacking ensemble methods and reported an accuracy of 86.7% and an AUC of 0.96 on the HAM10000 dataset. Similarly, Thwin and Park (2024) [39] employed an ensemble of VGG16, Inception-V3, and ResNet50, achieving up to 97% accuracy on a balanced ISIC dataset.

Compared to these methods, our model demonstrates competitive or superior performance, particularly in multiclass classification scenarios. Additionally, the integration of transformer-based and CNN-based models enables a hybrid feature extraction strategy that enhances generalization and robustness. Overall, this study lays a strong foundation for the development of intelligent, reliable, and accessible diagnostic systems for skin lesion analysis.

The key innovations of this work include: (1) A hybrid ensemble strategy that combines local and global feature extractors. (2) A focus on multiclass classification rather than binary melanoma detection. (3) Robust handling of class imbalance through static and dynamic data augmentation. (4) A design that is generalizable beyond a single dataset or population. These contributions provide a practical and scalable solution for computer-aided dermatological diagnosis, particularly in clinical settings where early and accurate detection is critical. In future work, we plan to conduct cross-dataset validation to assess the model’s ability to generalize across diverse demographic groups and clinical settings.

## Figures and Tables

**Table 2:** The number of cases for each of the 7 categories in the CSMUH dataset.

Class	Num	Label	Image
Malignant Melanoma	111	0	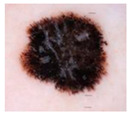
Melanocytic Nevus	199	1	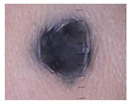
Basal cell carcinoma	45	2	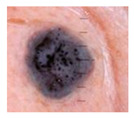
Actinic keratosis	82	3	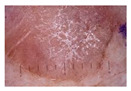
Benign keratosis	134	4	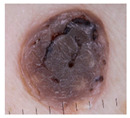
Dermatofibroma	42	5	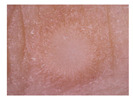
Vascular	53	6	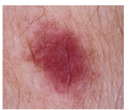

**Table 3:** Performance evaluation of Swin transformer on original and cropped datasets.

Dataset	Example	Model	Train ACC	Test ACC
Original	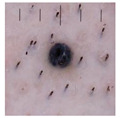	Swin base Transformer	0.991 ± 0.005	0.818 ± 0.046
cropped	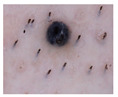		0.992 ± 0.005	0.845 ± 0.027

**Table 4:** Training and Testing Results of 25 Pretrained Models.

Architecture	No. of Models	Model	Train ACC	Test ACC
CNN	18	EfficientNetB1	0.993 ± 0.004	0.793 ± 0.050
		EfficientNetB2	0.960 ± 0.009	0.748 ± 0.015
		EfficientNetB3	0.989 ± 0.007	0.815 ± 0.027
		EfficientNetB4	0.993 ± 0.004	0.816 ± 0.016
		EfficientNetB5	0.991 ± 0.005	0.800 ± 0.021
		EfficientNetV2B2	0.965 ± 0.028	0.797 ± 0.036
		EfficientNetV2B3	0.985 ± 0.007	0.806 ± 0.015
		MobileNetV2	0.947 ± 0.020	0.749 ± 0.033
		DenseNet121	0.945 ± 0.038	0.736 ± 0.069
		DenseNet169	0.941 ± 0.058	0.748 ± 0.038
		DenseNet201	0.979 ± 0.016	0.785 ± 0.026
		VGGNet16	0.917 ± 0.038	0.685 ± 0.055
		Xception	0.981 ± 0.007	0.778 ± 0.034
		ResNet50	0.964 ± 0.017	0.787 ± 0.025
		InceptionV3	0.918 ± 0.049	0.733 ± 0.049
		InceptionResnetV2	0.973 ± 0.022	0.769 ± 0.051
		ConvNeXtSmall	0.947 ± 0.024	0.785 ± 0.042
		ConvNeXtBase	0.981 ± 0.023	0.806 ± 0.038
ViT	7	Vision b16 Transformer	0.995 ± 0.003	0.823 ± 0.022
		Vision b32 Transformer	0.996 ± 0.004	0.824 ± 0.032
		Vision l16 Transformer	0.995 ± 0.003	0.840 ± 0.023
		Vision l32 Transformer	0.997 ± 0.005	0.840 ± 0.012
		Swin small Transformer	0.987 ± 0.006	0.824 ± 0.023
		Swin base Transformer	0.992 ± 0.005	0.845 ± 0.027
		Swin large Transformer	0.991 ± 0.006	0.849 ± 0.022

**Table 5:** Comparison Results of Different Voting Ensemble Methods.

Voting Method	Architecture	Model	Train ACC	Test ACC
Hard voting	CNN	EfficientNetB3-EfficientNetB4-EfficientNetV2B3	0.999 ± 0.001	0.948 ± 0.017
	ViT	ViTb16-ViTl16-ViTl32	1.000 ± 0.000	0.954 ± 0.013
		SwinLarge-SwinBase-SwinSmall	0.995 ± 0.005	0.903 ± 0.022
	Ensemble	Swin-ViT-EfficientNetB4	0.999 ± 0.001	0.954 ± 0.020
Soft voting	CNN	EfficientNetB3-EfficientNetB4-EfficientNetV2B3	0.999 ± 0.001	0.952 ± 0.010
	ViT	ViTb16-ViTl16-ViTl32	1.000 ± 0.000	0.955 ± 0.013
		SwinLarge-SwinBase-SwinSmall	0.995 ± 0.005	0.921 ± 0.026
	Ensemble	Swin-ViT-EfficientNetB4	0.999 ± 0.001	0.961 ± 0.020

**Table 6:** Comparison Results of Different Ensemble Weights in Soft Voting.

Ensemble Weight (EfficientNetB4, ViT, Swin Base)	Train ACC	Test ACC
(1, 0, 0)	0.993 ± 0.004	0.816 ± 0.016
(0, 1, 0)	0.995 ± 0.003	0.840 ± 0.022
(0, 0, 1)	0.982 ± 0.018	0.824 ± 0.048
(1, 1, 0)	0.999 ± 0.002	0.952 ± 0.020
(1, 0, 1)	0.995 ± 0.009	0.937 ± 0.030
(0, 1, 1)	0.997 ± 0.004	0.954 ± 0.020
(1, 1, 1)	0.999 ± 0.001	0.961 ± 0.020

**Table 7:** Training Confusion Matrix of the Best Model.

	Predict							
True	label	0	1	2	3	4	5	6
	0	89	0	0	0	0	0	0
	1	0	159	0	0	0	0	0
	2	0	0	36	0	0	0	0
	3	0	0	0	65	0	0	0
	4	0	0	0	0	107	0	0
	5	0	0	0	0	0	34	0
	6	0	0	0	0	0	0	42

**Table 8:** Testing Confusion Matrix of the Best Model.

	Predict							
True	label	0	1	2	3	4	5	6
	0	22	0	0	0	0	0	0
	1	0	40	0	0	0	0	0
	2	0	1	8	0	0	0	0
	3	0	0	0	17	0	0	0
	4	0	0	0	0	26	0	0
	5	0	1	0	0	0	7	0
	6	0	0	0	0	0	0	11

**Table 9:** Testing Performance Indices of the Best Model.

Class	Sensitivity	Specificity	NPV	PPV	FPR	FNR	F1 Score
0	1	1	1	1	0	0	1
1	1	0.978	1	0.952	0.022	0	0.976
2	0.889	1	0.992	1	0	0.111	0.941
3	1	1	1	1	0	0	1
4	1	1	1	1	0	0	1
5	0.875	1	0.992	1	0	0.125	0.933
6	1	1	1	1	0	0	1

**Table 10:** A comparative result for the classification of seven types of skin lesions.

Author, Year	Method	Validation	Dataset	Class	Test ACC
[37], 2023	DenseNet121	Holdout (8:2) full: 666	CSMUH	7	0.851
	VGGNet16				0.731
	ResNet50				0.784
	MobileNet				0.813
	Xception				0.801
this study, 2025	Ensemble (vote) (Swin, ViT, EfficientNetB4)	Holdout (8:2) full: 666	CSMUH	7	0.985

## Data Availability

The CSMUH data that support the findings of this study are available from the corresponding author upon reasonable request.
